# Early postoperative serum IL-6 may contribute to risk stratification for pancreas-specific complications after pancreatic resection

**DOI:** 10.1007/s00423-026-04283-0

**Published:** 2026-09-14

**Authors:** Hanna Schulte-Hürmann, Faruk Koca, Julia Wicke, Elena Mazzella, Tamás Benkö, Armin Wiegering, Ekaterina Petrova

**Affiliations:** Department of General, Visceral, Transplant and Thoracic Surgery, University Hospital, Goethe University Frankfurt, Theodor-Stern-Kai 7, Frankfurt, 60590 Germany

**Keywords:** IL-6, Postpancreatectomy acute pancreatitis, Postoperative pancreatic fistula, Pancreas

## Abstract

**Purpose:**

IL-6 level in serum has been shown to have predictive value for postoperative infectious complications and prognostic value in acute pancreatitis. Evidence on its role after pancreatic resection is limited. The aim of the study was to analyse the predictive role of early postoperative serum IL-6 regarding complications after pancreatic resection.

**Methods:**

A retrospective analysis of patients with pancreatic resection at a tertiary German centre was performed. All IL-6 values per patient within 48 h after resection were delineated. Values at fixed postoperative time points were interpolated between each patient’s measurements and, beyond them, predicted from a linear mixed-effects model. Receiver operating characteristic curve analysis was performed, and optimal time and cut-off points for prediction of postoperative pancreatic fistula (POPF), postpancreatectomy acute pancreatitis (PPAP) and overall complications were set out.

**Results:**

Out of 316 patients, 157 patients with more than one IL-6 value were included. IL-6 at 36 h after resection showed the best diagnostic performance for all three endpoints. For PPAP B/C, the AUC was 0.800 (95% CI 0.685–0.916) at a cut-off of 296 pg/ml (sensitivity 62%, specificity 80%, PPV 26%, NPV 95%), and for POPF B/C 0.715 (0.632–0.798) at 192 pg/ml (sensitivity 70%, specificity 59%, PPV 52%, NPV 76%). Discrimination for major complications (Clavien–Dindo ≥ 3b) was weaker, with an AUC of 0.636 (0.539–0.733) at 208 pg/ml (sensitivity 55%, specificity 62%, PPV 44%, NPV 73%).

**Conclusion:**

Serum IL-6 within 48 h after pancreatic resection could be useful in risk assessment for pancreas-specific complications.

**Supplementary Information:**

The online version contains supplementary material available at https://doi.org/10.1007/s00423-026-04283-0.

## Introduction

Postoperative pancreatic fistula (POPF) is a characteristic complication of pancreatic resection associated with substantial postoperative morbidity and mortality [1, 2]. In recent years, postpancreatectomy acute pancreatitis (PPAP) has been recognized as a distinct process that poses a risk on its own and that might lead to a secondary leak of the pancreatic anastomosis and thus POPF [3]. Biomarkers are of interest for prognostication, understanding the natural course and designing potential interventions to prevent POPF and PPAP. Interleukin-6 (IL-6) is a cytokine with a broad spectrum of biological functions, including roles in inflammation, oncogenesis and immunomodulation [4]. IL-6 is predictive of postoperative infectious complications after abdominal surgery [5]. Further, elevated IL-6 levels in serum have been shown to have a prognostic role in acute pancreatitis during the first 48 h after onset [6–8]. The role of serum IL-6 after pancreatic resection, especially regarding prediction of pancreas-specific complications, has hardly been studied. In a side study of the multicenter randomized controlled LEOPARD-2 trial, which compared laparoscopic and open pancreatoduodenectomy, van Hilst et al. showed higher IL-6 levels in patients with complications Clavien-Dindo Classification (CDC) grade ≥ 3 and POPF B/C according to the classification of the International Study Group for Pancreatic Surgery (ISGPS) [9]. In a prospective study on 70 patients after pancreatic resection, Gasteiger et al. demonstrated that elevated lipase and IL-6 on the third postoperative day were predictive of POPF and overall complications [10].

In this study, we aimed to analyze the predictive value of serum IL-6 during the first 48 h after pancreatic resection, regarding POPF, PPAP and overall postoperative complications.

## Materials and methods

### Study design

This is a retrospective, explorative, single-center study. It was approved by the ethics committee of the University Hospital, Goethe University Frankfurt, Germany (Ref. 2023 − 1428).

### Patients and Data

All consecutive patients with pancreatic resection at the University Hospital, Goethe University Frankfurt, from January 2016 to April 2023 were included. Patients with total pancreatectomy were excluded. Demographic and clinical data were collected retrospectively from the electronic patient records. Laboratory data, including all serum values of IL-6, lipase and amylase, as well as white blood cell count (WBC), obtained within 48 h after the end of the operation, were extracted. The end of the operation was defined as the end of wound closure. Sampling followed clinical routine, and no fixed sampling schedule existed, so the number and timing of measurements varied between patients.

Patient records, laboratory data and postoperative imaging were reviewed to detect patients with POPF B/C or PPAP B/C. POPF B/C was defined and classified according to the ISGPS 2016 update [2] At our institution, serum lipase rather than amylase is measured routinely in the early postoperative period. In the prospective multicentre validation of the ISGPS definition, post-pancreatectomy hyperenzymaemia was defined by sustained elevation of serum amylase or lipase on postoperative days 1 and 2, and the applicability of serum lipase for this purpose was confirmed [11]; PPAP was therefore classified using serum lipase. Postoperative complications were categorized according to the Clavien-Dindo Classification [12] Outcome parameters analyzed were POPF B/C, PPAP B/C and major postoperative complications (CDC grade ≥3b). Laboratory measurements were conducted as part of routine clinical care. For IL-6 the Roche ECLIA (Electrochemiluminescence) platform with normal reference range < 7 pg/ml was used.

### Statistical Analysis

Statistical analysis was done with R Version 4.5.1 [13] Descriptive analysis with absolute numbers and proportion of total for binary and categorical variables as well as median and interquartile range (IQR) for continuous variables was performed. Patients with fewer than two IL-6 values within 48 h after resection were excluded. To account for a potential selection bias, these patients were compared to the patients with two or more IL-6 values present, using the Kruskal-Wallis or χ2-Test, as appropriate.

Median values of IL-6 (IL-6_m_), lipase (lipase_m_) and WBC (WBC_m_) within 48 h post resection were calculated. Amylase was not further considered due to the large proportion of missing data. The minimum (IL-6_min_) and maximum (IL-6_max_) IL-6 values and their corresponding time points were delineated. Values at the fixed time points 6, 12, 18, 24, 30, 36 and 42 h postoperatively were obtained by linear interpolation between each patient’s own measurements; where a time point fell before the first or after the last measurement of that patient, the value was predicted from a linear mixed-effects model. Because more than 70% of values at 42 h would have required extrapolation beyond the last available measurement in all three markers, this time point was excluded and all further analyses were restricted to 60–36 h postoperatively. Leave-one-measurement-out cross-validation was performed to assess the accuracy of the estimates. Details of the value estimation are given in the Supplementary Figure S1.

Spearman rank correlations between the laboratory parameters were calculated (Supplementary Figure S2). Univariable analysis with the Kruskal-Wallis test was performed to detect differences in median IL-6, lipase and WBC across the levels of binary or categorical clinical variables. Associations between clinical parameters and the postoperative IL-6 level were analysed in a multivariable linear regression with the per-patient median IL-6 concentration as the dependent variable. Because IL-6 concentrations were right-skewed, the values were log-transformed; model assumptions were checked by inspection of residual and quantile-quantile plots. Type of resection, age, histology, sex, BMI and main pancreatic duct diameter were entered simultaneously, without variable selection. Five patients with unknown main pancreatic duct diameter were excluded from this model. Pancreatic texture was not included because of the high proportion of missing values.

Receiver operating characteristic (ROC) curve analysis was performed with the pROC R package to compare the prognostic value of each of the laboratory parameters for each of the outcome parameters [14]. In the case of PPAP, lipase was not further considered, since the PPAP definition is based on the serum lipase level. Optimal cut-off values were calculated by finding the closest point to the top-left corner (“closest topleft” method). To account for potential overfitting and model optimism, the entire analysis — including model fitting, reconstruction of the missing time points, cut-off selection and calculation of all diagnostic measures — was repeated in 500 bootstrap samples drawn at the patient level, and the average difference between bootstrap and original performance was subtracted from the apparent estimates [15]. All performance measures including area under the curve (AUC), sensitivity, specificity, positive predictive value (PPV) and negative predictive value (NPV) reported in the final analysis are optimism-corrected. The median optimal cut-off value across all bootstrap samples was calculated and presented. Comparison of apparent and optimism-corrected values is given in the Supplementary Table S6. The laboratory parameters with the highest AUC were dichotomized according to the cut-off values and included in a multivariable logistic regression model. As a sensitivity analysis, the diagnostic performance was recalculated in the subgroup of patients whose own measurement window extended to the respective time point, so that no extrapolated values entered this analysis.

## Results

Overall, 316 patients were identified. Of those, two or more IL-6 values within 48 h after resection were available in 157 patients. Descriptive statistics of the whole cohort as well as univariable comparison of patients with two or more and those with fewer than two IL-6 values are displayed in the Supplementary Tables S1 and S2, respectively. Among the patients with two or more IL-6 values, there were significantly more patients with pancreatic head resection (87.9% vs. 74.8%) compared to distal pancreatectomy (12.1% vs. 25.2%, *p* = 0.005). Correspondingly, the difference in median operation time was also significant (233 vs. 207 min, *p* = 0.001). There were no other statistically significant differences.

Descriptive statistics of the subgroup with two or more IL-6 values are displayed in Table 1. Median age at the time of resection was 68 years (IQR 58, 77). There was a slight predominance of males (58%). There were 138 (88%) patients with pancreatic head resection, among them 116 with pylorus-preserving pancreatoduodenectomy, 20 with pylorus-resecting pancreatoduodenectomy, 2 with duodenum preserving pancreatic head resection; 19 (12%) patients had distal pancreatic resection. The most common diagnosis was pancreatic ductal adenocarcinoma (PDAC) in 72 (46%) patients. The rate of POPF B/C was 39%, of PPAP B/C 10%, and of major complications (CDC ≥ 3b) 34%.

**Fig. 1:**
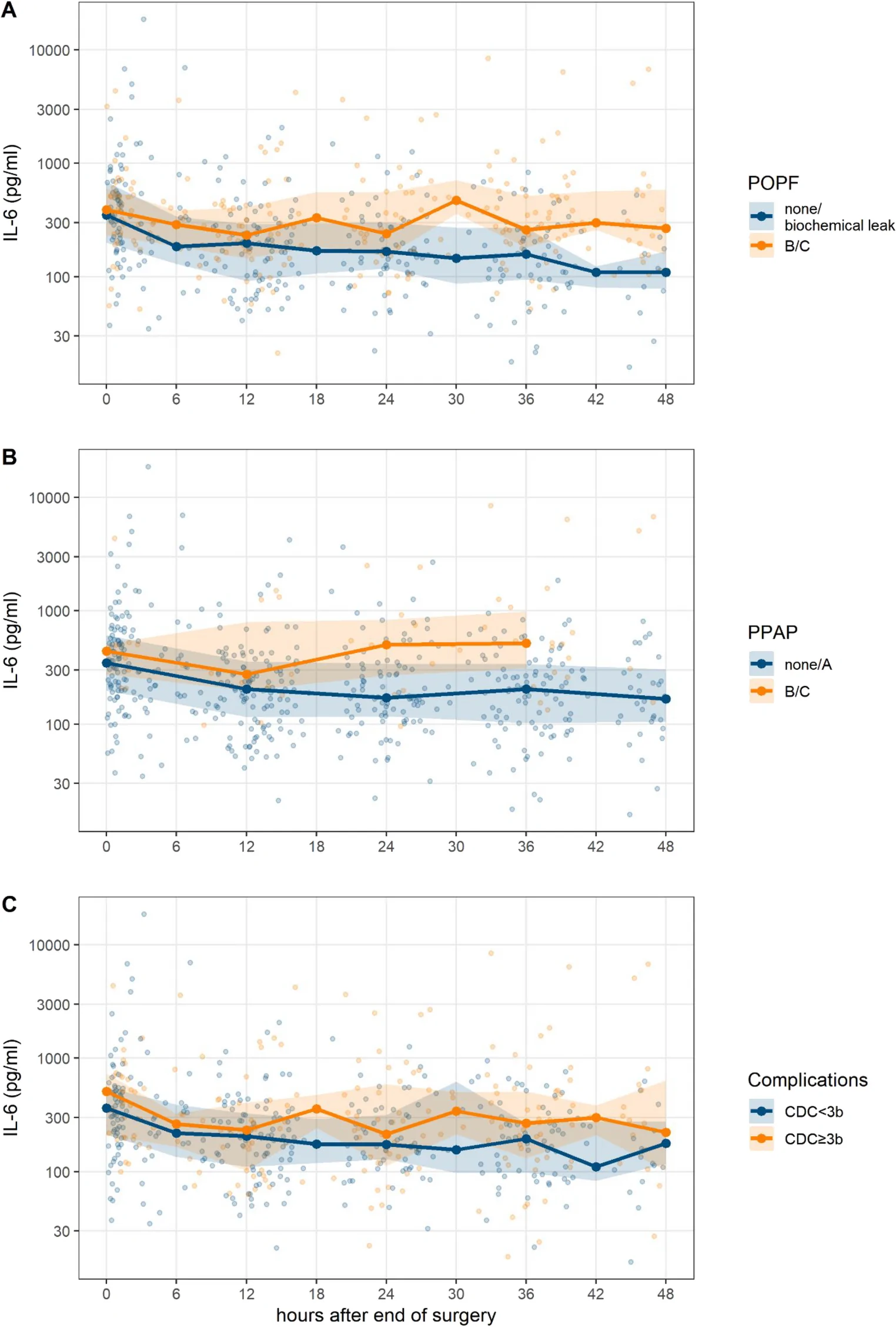
Time course of measured IL-6 levels within 48 h after surgery stratified for (**A**) postoperative pancreatic fistula (POPF), (**B**) postpancreatectomy acute pancreatitis (PPAP), (**C**) Overall complications Clavien-Dindo Classification (CDC) Grade ≥ 3b. Faint points represent single measurements; lines show the median with interquartile range within six-hour intervals (twelve hours in panel B). Concentrations are shown on a logarithmic scale

For each patient there were a median of 3 (IQR 2, 4) values within 48 h after resection. Patients who developed POPF B/C had more IL-6 measurements than those who did not (median 4 versus 3, *p* = 0.014). The time course of measured IL-6 levels stratified by POPF, PPAP and overall complications is displayed in Fig. 1. For each of the time points between 6 and 36 h, the proportion of values falling within a patient’s own measurement window ranged from 52% to 72% for IL-6, 62% to 86% for lipase and 78% to 90% for WBC, whereas at 42 h only 11% to 29% of values were covered; this time point was therefore excluded from the further analysis (Supplementary Table S4). An IL-6 value at or beyond 36 h was available in 44 of 61 patients with POPF B/C (72%) compared with 45 of 96 without (47%; *p* = 0.003).

The overall median IL-6 value was 230 pg/ml (IQR 138–384). The median time of the first IL-6 value measured was 2 h (IQR 1, 8) and the median time of the last value measured was 37 h (IQR 15, 40) after the end of the operation. The median IL-6_max_ value was 405 pg/ml (IQR 238, 689) and was reached at 6 h (IQR 1, 25). The median IL-6_min_ value was 145 pg/ml (IQR 81, 259) and was reached at 21 h (IQR 12, 38). The median lipase value within 48 h after surgery (lipase_m_) was 60 U/l (IQR 16, 141), the median WBC_m_ was 13.4/nl (IQR 10.6, 16.3; Table 1). There was a strong correlation between IL-6_max_ and IL-6_6_ (Spearman’s ρ = 0.90; Supplementary Figure S2).

In the univariable analysis, patients older than 70 years, histology other than PDAC or chronic pancreatitis (CP), and pancreatic head resection had significantly higher IL-6_m_. Patients with splenectomy had lower IL-6_m_ whereas, patients with a bile duct stent prior to resection had higher IL-6_m_. IL-6_m_ was significantly higher in patients with POPF B/C, PPAP B/C and CDC≥3b (Supplementary Table S3). Of the variables entered into the multivariable model, only age above 70 years (β 0.46, 95% CI 0.18, 0.74; *p* = 0.001) and a histology other than PDAC or CP (β 0.39, CI 0.09, 0.69; *p* = 0.012) remained associated with higher IL-6_m_ (Table 2).

**Fig. 2:**
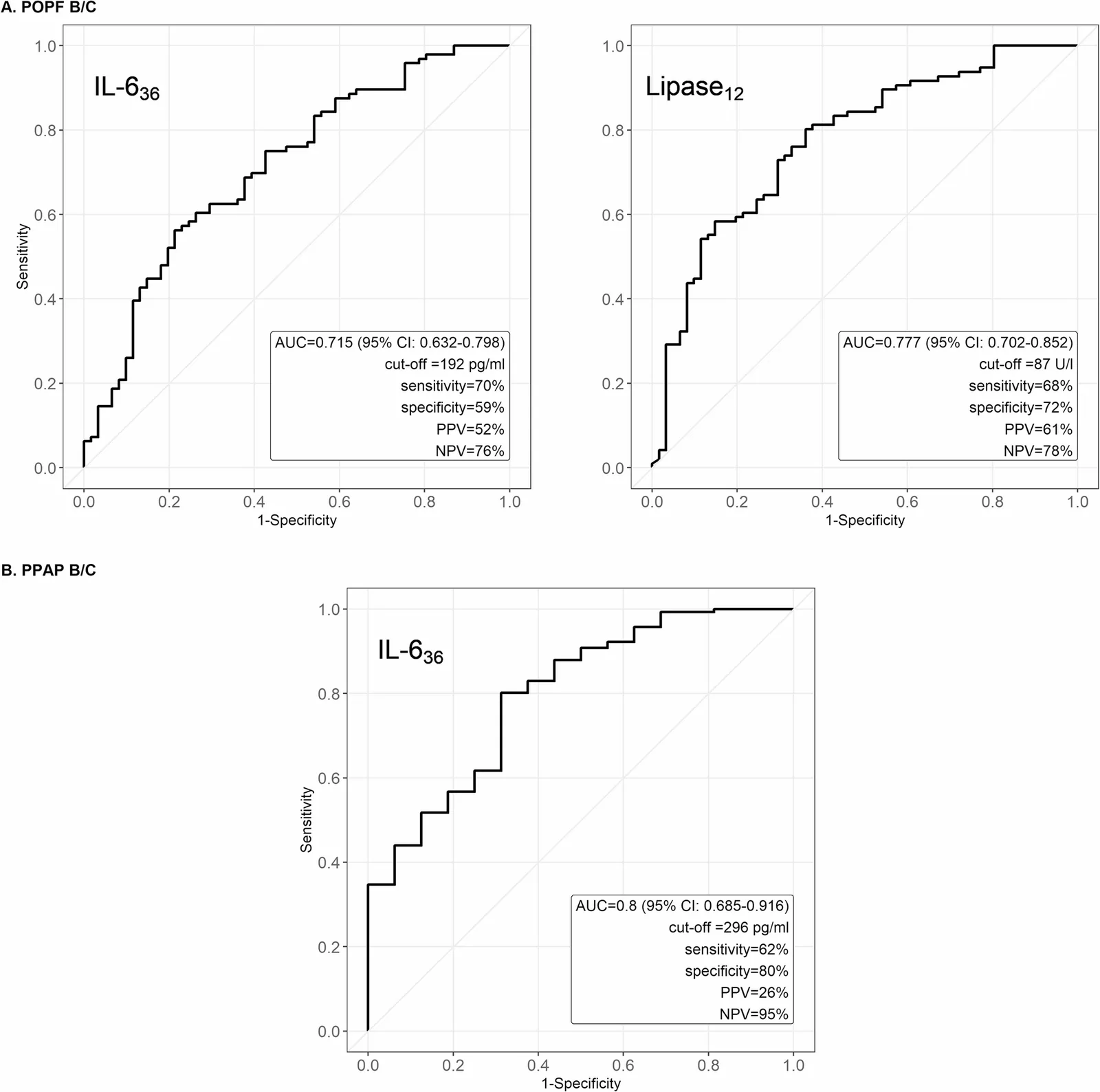
Receiver Operating Characteristic (ROC) curves for (**A**) postoperative pancreatic fistula (POPF) B/C and (**B**) postpancreatectomy acute pancreatitis (PPAP) B/C

In the ROC analysis for POPF B/C, lipase at 12 h (lipase₁₂) showed the best discrimination (Fig. 2; AUC 0.777, 95% CI 0.702, 0.852; cut-off 87.1 U/l; sensitivity 68%, specificity 72%, PPV 61%, NPV 78%). Among the IL-6 time points, IL-6₃₆ performed best (Fig. 2; AUC 0.715, CI 0.632, 0.798; cut-off 191.8 pg/ml; sensitivity 70%, specificity 59%, PPV 52%, NPV 76%), with discrimination increasing steadily from 6 to 36 h. WBC discriminated poorly at all time points (AUC ≤ 0.64).

For PPAP B/C, IL-6₃₆ showed the highest discrimination (Fig. 2; AUC 0.800, 0.685–0.916; cut-off 296.1 pg/ml; sensitivity 62%, specificity 80%, PPV 26%, NPV 95%). From 18 h onwards, all IL-6 time points reached an NPV of 95%. WBC did not discriminate (AUC ≤ 0.571).

For major postoperative complications (CDC ≥ 3b), discrimination was weaker, with IL-6₃₆ again performing best (AUC 0.636, 0.539–0.733; cut-off 208.3 pg/ml; sensitivity 55%, specificity 62%, PPV 44%, NPV 73%). The complete ROC analysis is presented in the Supplementary Figures S3–S5.

In a sensitivity analysis restricted to patients with a measurement at or beyond 36 h (*n* = 89), IL-6₃₆ yielded an AUC of 0.764 (95% CI 0.664–0.864) for POPF B/C and 0.770 (0.630–0.911) for PPAP B/C.

**Fig. 3:**
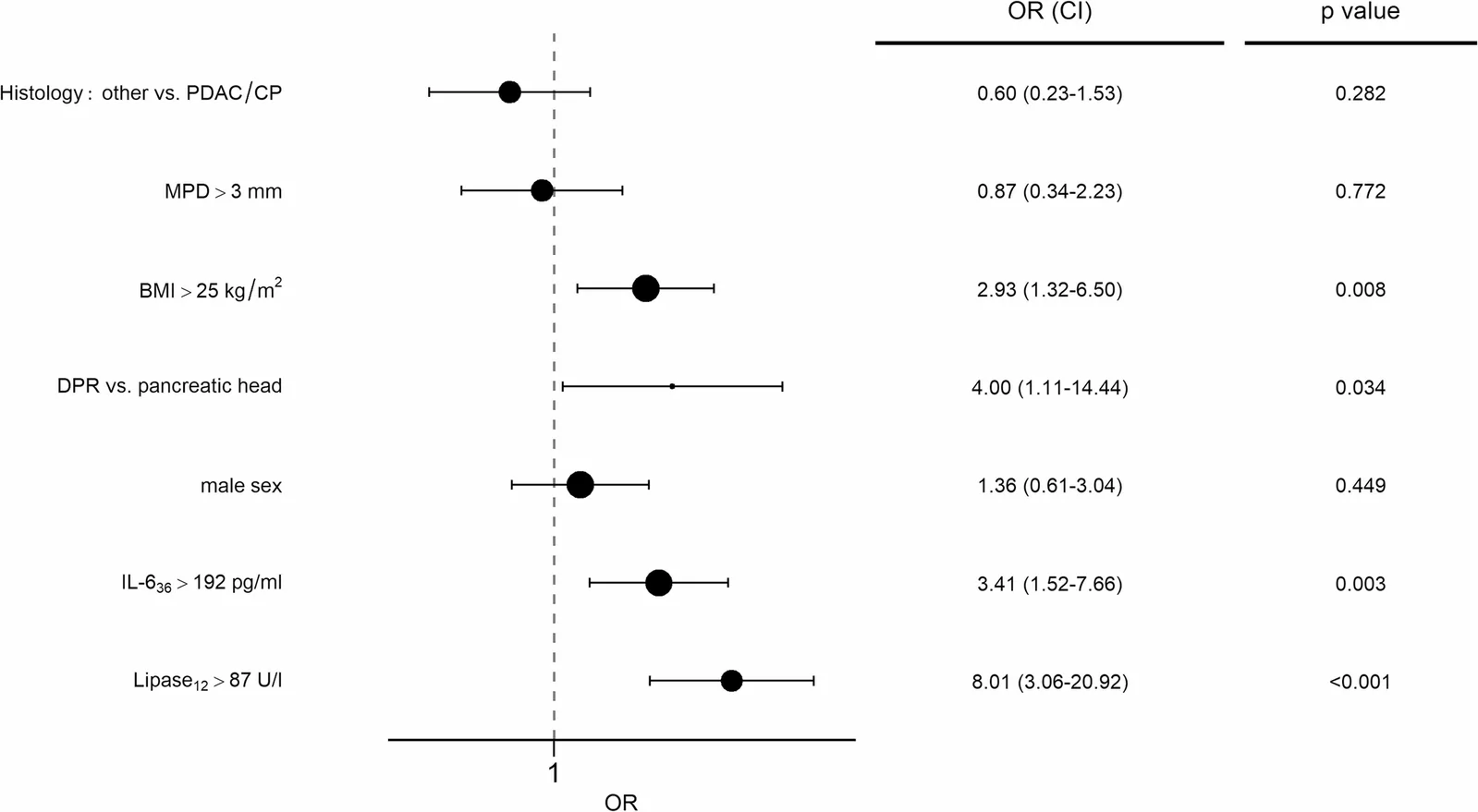
Multivariable logistic regression for postoperative pancreatic fistula (POPF) B/C. Five patients with unknown main pancreatic duct diameter were excluded from the model (*n* = 152 of 157)

In the multivariable logistic regression for POPF B/C, lipase₁₂ > 87 U/l (OR 8.01, 95% CI 3.06–20.92; *p* < 0.001), distal pancreatectomy compared with pancreatic head resection (OR 4.00, 1.11–14.44; *p* = 0.034), IL-6₃₆ > 192 pg/ml (OR 3.41, 1.52–7.66; *p* = 0.003) and BMI > 25 kg/m² (OR 2.93, 1.32–6.50; *p* = 0.008) were independently associated with the outcome. Male sex (OR 1.36, 0.61–3.04; *p* = 0.449), a main pancreatic duct diameter > 3 mm (OR 0.87, 0.34–2.23; *p* = 0.772) and histology other than PDAC or chronic pancreatitis (OR 0.60, 0.23–1.53; *p* = 0.282) were not associated with POPF B/C. All model terms are shown in Fig. 3.

## Discussion

In this retrospective study, we analysed the temporal dynamics and predictive value of serum IL-6 levels in the first 48 h after pancreatic resection. We demonstrated that serum IL-6 peaked at about 6 h after the end of the operation and fell to a minimum at about 21 h. Median IL-6 level was higher in patients with POPF B/C, PPAP B/C and major complications CDC ≥ 3b within the first 48 h after resection. Our data suggest that early serum IL-6 kinetics can possibly identify patients at low risk for developing clinically relevant PPAP, and that IL-6 together with early serum lipase does so for POPF B/C.

Regarding PPAP B/C, IL-6₃₆ showed the highest discrimination with an AUC of 0.800 (95% CI 0.685–0.916); at a cut-off of 296.1 pg/ml, sensitivity was 62%, specificity 80%, PPV 26% and NPV 95%. Notably, all values at time points 18 to 36 yielded an NPV of 95%.

For POPF B/C, the diagnostic performance of IL-6 was moderate but chronologically distinct. Il-6_36_ showed the best overall discrimination (AUC 0.715); at the optimal cut-off of 191.8 pg/ml sensitivity was 70% and NPV 76%. This may indicate that later timepoints of IL-6 monitoring are more reflective of the ongoing inflammatory response associated with a developing fistula. However, when comparing biomarkers for POPF B/C, postoperative serum lipase showed the numerically highest discrimination with an AUC of 0.777 at 12 h; at a cut-off of 87.1 U/l, sensitivity was 68%, specificity 72%, and NPV 78%. In multivariable analysis, lipase₁₂ and IL-6₃₆ remained independent predictors of POPF B/C, highlighting the potential clinical utility of combining both markers.

Postoperative IL-6 serum levels have been demonstrated to be useful in detecting infectious complications in abdominal surgery. Compared to other markers, like C-reactive protein (CRP), IL-6 increases earlier and thus allows for a rapid intervention [16, 17]. In this study, we saw higher median postoperative IL-6 levels in patients with overall complications CDC ≥3b, but the predictive value was low with highest AUC 0.636 in the ROC analysis for IL-6_36_. Our analysis suggests that early IL-6 in serum after pancreatic resection has a better predictive value for the pancreas-specific complications POPF B/C and PPAP B/C. Further, higher postoperative serum lipase is an independent predictor for POPF B/C, which is in concordance with the results of Gasteiger et al. While Gasteiger et al. showed this for the third postoperative day, we restricted our analysis to the first 48 h [10]. This rationale is supported by previous studies demonstrating that IL-6 possesses prognostic value within the first 48 h of acute pancreatitis onset, as well as on the first postoperative day following major abdominal surgery to predict major complications [7, 17].

**Table 1:** Descriptive statistics of the cohort with two or more IL-6 values (*n* = 157)

Parameter	Median (Q1, Q3); *n* (%)
Age	68 (58, 77)
BMI [kg/m²]	24.3 (22.0, 28.7)
Sex	
Female	66 (42%)
Male	91 (58%)
Type of resection	
PRPD	20 (13%)
PPPD	116 (74%)
DPR	19 (12%)
DPPHR	2 (1.3%)
Histology	
PDAC	72 (46%)
DBDC	17 (11%)
AMPAC	16 (10%)
DUODAC	10 (6.4%)
NET	12 (7.6%)
IPMN	3 (1.9%)
CP	13 (8.3%)
other	14 (8.9%)
Antibiotics prior to resection	7 (4.5%)
WBCₚ_r_ₑ [/nl]	7.33 (5.77, 8.60)
CRPₚ_r_ₑ [mg/dl]	0.39 (0.19, 1.20)
unknown	23
Lipaseₚ_r_ₑ [U/l]	50 (29, 83)
unknown	27
operation time [min]	233 (195, 282)
splenectomy	17 (11%)
PVR	12 (7.6%)
Parenchyma	
Soft	55 (35%)
Hard	44 (28%)
Unknown	58 (37%)
MPD	
≤ 3 mm	99 (63%)
> 3 mm	53 (34%)
unknown	5 (3.2%)
Bile duct stent	38 (24%)
Antibiotics 24 h post resection	76 (48%)
POPF B/C	61 (39%)
DGE B/C	43 (27%)
PPH B/C	36 (23%)
PPAP B/C	16 (10%)
LOS [days]	19 (12, 28)
30-day reoperation rate	35 (22%)
major complications CDC ≥ 3b	54 (34%)
number of IL-6 values	3 (2, 4)
IL-6ₘ [pg/ml]	230 (138, 384)
IL-6ₘₐₓ [pg/ml]	405 (238, 689)
tₘ IL-6ₘₐₓ [h]	6 (1, 25)
IL-6ₘ_i_ₙ [pg/ml]	145 (81, 259)
tₘ IL-6ₘ_i_ₙ [h]	21 (12, 38)
tₘ IL-6_first_ [h]	2 (1, 8)
tₘ IL-6ₗₐₛₜ [h]	37 (15, 40)
Lipaseₘ [U/l]	60 (16, 141)
WBCₘ [/nl]	13.4 (10.6, 16.3)

Abbreviations: *AMPAC* ampullary carcinoma, *BMI* body mass index, *CDC* Clavien-Dindo Classification, *CP* chronic pancreatitis, *CRP* C-reactive protein, *DBDC* distal bile duct carcinoma, *DGE* delayed gastric emptying, *DPR* distal pancreatectomy, *DPPHR* duodenum-preserving pancreatic head resection, *DUODAC* duodenal carcinoma, *h* hours, *IL-6* interleukin-6, *IPMN* intraductal papillary mucinous neoplasia, *LOS* length of stay, __*m*_ median, *max* maximal, *min* minimal, *MPD* main pancreatic duct diameter, *NET* neuroendocrine tumor, *PDAC* pancreatic ductal adenocarcinoma, *POPF* postoperative pancreatic fistula, *PPAP* postpancreatectomy acute pancreatitis, *PPH* postpancreatectomy hemorrhage, *PPPD* pylorus-preserving pancreatoduodenectomy, _*_pre*_ preoperative, *PRPD* pylorus-resecting pancreatoduodenectomy, *PVR* portal vein resection, *t*_*m*_ median time, *WBC* white blood cell count

Due to institutional clinical protocols, postoperative CRP was not routinely measured within the 48-hour window when IL-6 was monitored, as CRP typically exhibits a delayed kinetic peak. Thus, a direct comparison between CRP and IL-6 in this case was not possible. Instead, we used WBC count—which is routinely measured postoperatively and used as an inflammation marker—as a comparative baseline. The predictive power of WBC in our cohort was low, supporting the added value of early IL-6 monitoring.

While in some institutions IL-6 might routinely be used as a marker after major abdominal surgery, our findings suggest that in the case of pancreatic resection marked elevations within the first 48 h are more closely associated with pancreas-specific complications than with overall major morbidity. Due to the retrospective design and the relatively small cohort size, patients with concurrent infectious complications were not excluded from this analysis. However, in real-world clinical practice, distinguishing between a systemic inflammatory response syndrome and the onset of a surgical complication during this early phase remains highly complex. According to the ISGPS consensus definitions, PPAP develops early and is defined within the first 24 to 48 h, while POPF is formally diagnosed from postoperative day 3 onwards based on amylase levels in the drain fluid. Consequently, our IL-6 measurements taken within the first 48 h capture the acute, initial stages of these distinct pathophysiological entities. To distinguish retrospectively whether an elevation in IL-6 was driven by reaction to other early infectious complications, involving possible reverse causality with pancreas-specific complications, is nearly impossible. Therefore, these results must be interpreted purely as associative rather than causal.

**Table 2:** Multivariable linear regression for the per-patient median IL-6 concentration within 48 h after resection (*n* = 152)

Parameter	Beta*	95% CI	*p*-value
Type of resection			
Pancreatic head	—	—	
DPR	-0.43	-0.87, 0.01	0.056
Age			
≤ 70	—	—	
> 70	0.46	0.18, 0.74	0.001
Histology			
PDAC/CP	—	—	
other	0.39	0.09, 0.69	0.012
Sex			
female	—	—	
male	0.18	-0.10, 0.46	0.215
BMI [kg/m²]			
≤ 25	—	—	
> 25	0.02	-0.26, 0.30	0.883
MPD			
≤ 3 mm	—	—	
> 3 mm	0.06	-0.25, 0.37	0.700

*Coefficients refer to log-transformed IL-6 concentrations.

Five patients with unknown main pancreatic duct diameter were excluded from the model (*n* = 152 of 157).

Abbreviations: *BMI *body mass index, *DPR* distal pancreatectomy, *CI* confidence interval, *CP* chronic pancreatitis, *MPD* main pancreatic duct diameter, *PDAC* pancreatic ductal adenocarcinoma

An important methodological limitation of this study is the inclusion criterion requiring at least two distinct IL-6 measurements within the first 48 h. While this approach was necessary to capture the dynamic kinetics of IL-6, it resulted in the exclusion of more than half of the initial cohort (reducing the sample size from 316 to 157 patients). The remaining cohort was heterogeneous, with pancreatic head resections predominating over distal pancreatectomies. The longer operative times observed in the IL-6 cohort are likely a direct clinical consequence of the higher proportion of pancreatic head resections within this group. This may have introduced a selection bias, as the included patients had higher perceived perioperative risk that warranted closer monitoring in intermediate or intensive care settings, which limits the generalizability of our findings. Furthermore, pooling patients undergoing pancreatic head resections and distal pancreatectomies may fail to capture the procedure-specific nuances of PPAP and POPF.

Because sampling followed clinical routine, values at the analysed time points were not available in every patient and were reconstructed by linear interpolation within each patient’s measurement window and by a mixed-effects model beyond it. In leave-one-measurement-out validation, the median absolute percentage error for IL-6 was 24.3% within the measurement window versus 45.6% and 33.0% before the first and after the last available measurement, with median predicted/observed ratios of 0.99–1.10. Because IL-6 has a short half-life and can change several-fold within hours — values in our cohort ranged from 16 to 18,629 pg/ml — an error of 45% corresponds to a deviation of about 1.5-fold, which is modest on this scale but too coarse to reconstruct individual values far outside a patient’s sampled interval. As more than 70% of patients would have required extrapolation at 42 h, this time point was dropped. Patients who developed POPF B/C had more IL-6 measurements than those who did not, and a late measurement was more often available in this group — an expected consequence of clinical routine, in which deterioration prompts closer monitoring. Values at 36 h were therefore more often extrapolated in patients without a fistula, so the reconstruction was not equally accurate in both groups. In a sensitivity analysis restricted to patients with a measurement at or beyond 36 h, discrimination was similar to that in the full cohort, indicating that the reconstruction did not generate the observed associations.

The total event count for PPAP B/C was small (16 patients). This low incidence inherently restricts the statistical power and generalizability of our predictive metrics for this specific complication, highlighting the exploratory nature of our findings and the need for larger prospective validation. Further, due to a high rate of missing values, pancreatic texture could not be incorporated into a standardized ISGPS risk classification or included in the final multivariable regression model without severely compromising statistical power.

In conclusion, this study suggests that elevated IL-6 serum levels within 48 h after pancreatic resection are associated with clinically relevant postoperative pancreatic fistula and especially postpancreatectomy acute pancreatitis, with early serum lipase adding predictive information for POPF. These exploratory findings require validation in independent patient cohorts to confirm their clinical utility.

## Supplementary Information

Below is the link to the electronic supplementary material.

Supplementary Material 1 (DOCX 1.11 MB)

## Data Availability

The data that support the findings of this study are not openly available due to reasons of sensitivity and are available from the corresponding author upon reasonable request.
