# Supplementary material for: Early postoperative serum IL-6 may contribute to risk stratification for pancreas-specific complications after pancreatic resection
Source: Langenbecks Arch Surg. 2026 Sep 14;411(1):235. doi: 10.1007/s00423-026-04283-0 (PMC13578034; doi:10.1007/s00423-026-04283-0)
Supplement: Supplementary file 1 [file 423_2026_4283_MOESM1_ESM.docx]

Supplementary

**Table S1: Descriptive statistics for the overall cohort (N=316)**

| **Parameter** | **Median (Q1, Q3); n (%)** |
| --- | --- |
| age | 68 (58, 76) |
| sex |  |
| female | 132 (42%) |
| male | 184 (58%) |
| BMI [kg/m²] | 24.6 (22.3, 27.9) |
| type of resection |  |
| PRPD | 32 (10%) |
| PPPD | 221 (70%) |
| DPR | 59 (19%) |
| DPPHR | 4 (1.3%) |
| histology |  |
| PDAC | 141 (45%) |
| DBDC | 28 (8.9%) |
| AMPAC | 24 (7.6%) |
| DUODAC | 24 (7.6%) |
| NET | 26 (8.2%) |
| IPMN | 15 (4.7%) |
| CP | 27 (8.5%) |
| other | 31 (9.8%) |
| bile duct stent | 65 (21%) |
| antibiotics prior to resection | 9 (2.8%) |
| WBCₚᵣₑ [/nl] | 7.45 (5.78, 8.91) |
| unknown | 1 |
| CRPₚᵣₑ [mg/dl] | 0.46 (0.20, 1.46) |
| unknown | 71 |
| Lipaseₚᵣₑ [U/l] | 48 (29, 87) |
| unknown | 47 |
| operation time [min] | 222 (184, 271) |
| splenectomy | 49 (16%) |
| PVR | 22 (7.0%) |
| parenchyma |  |
| soft | 107 (34%) |
| hard | 85 (27%) |
| unknown | 124 (39%) |
| MPD |  |
| ≤3mm | 193 (61%) |
| >3mm | 112 (35%) |
| unknown | 11 (3.5%) |
| antibiotics 24 hours post resection | 139 (44%) |
| IL-6ₘ [pg/ml] | 229 (132, 384) |
| unknown | 142 |
| Lipaseₘ [U/l] | 59 (16, 133) |
| unknown | 1 |
| WBCₘ [/nl] | 13.3 (10.6, 16.1) |
| POPF B/C | 108 (34%) |
| DGE B/C | 64 (20%) |
| PPH B/C | 66 (21%) |
| PPAP B/C | 28 (8.9%) |
| LOS [days] | 16 (11, 27) |
| 30-day reoperation rate | 62 (20%) |
| ICU readmission rate | 96 (30%) |
| major complications rate CDC≥3b | 104 (33%) |
| *Abbreviations: AMPAC – ampullary carcinoma, BMI – body mass index, CDC – Clavien-Dindo Classification, CP – chronic pancreatitis, CRP – C-reactive protein, DBDC – distal bile duct carcinoma, DGE – delayed gastric emptying; DPR – distal pancreatectomy, DPPHR – duodenum-preserving pancreatic head resection, DUODAC – duodenal carcinoma, ICU – intensive care unit, IL-6 – interleukin 6, IPMN – intraductal papillary mucinous neoplasia, LOS – length of stay, __m_ – median, MPD – main pancreatic duct diameter, NET – neuroendocrine tumor, PDAC – pancreatic ductal adenocarcinoma, POPF – postoperative pancreatic fistula, PPAP – postpancreatectomy acute pancreatitis, PPH – postpancreatectomy hemorrhage, PPPD – pylorus-preserving pancreatoduodenectomy, __pre_ – preoperative, PRPD – pylorus-resecting pancreatoduodenectomy, PVR – portal vein resection, WBC – white blood cell count* | |

**Table S2: Univariable comparison of patients with 2 or more IL-6 values and those with fewer than 2 values 48 hours after resection.**

| **Parameter** | **≥2 IL-6 values** | | **<2 IL-6 values** | **p-value** |
| --- | --- | --- | --- | --- |
| age | | 68 (58,77) | 68 (58,75) | 0.328 |
| male sex | | 91 (58) | 93 (58.5) | >0.99 |
| BMI [kg/m²] | | 24.3 (22,28.7) | 25 (22.5,27.4) | 0.880 |
| WBC_pre_ [/nl] | | 7.3 (5.8,8.6) | 7.6 (5.8,9) | 0.375 |
| CRP_pre_ [mg/dl] | | 0.4 (0.2,1.2) | 0.6 (0.2,1.7) | 0.131 |
| Lipase_pre_ [U/l] | | 50 (29,82.8) | 45 (29.5,88.5) | 0.716 |
| operation time [min] | | 233 (195,282) | 207 (172.5,253) | 0.001 |
| Lipase_m_ [U/l] | | 60 (16,141) | 55.3 (16.8,115.5) | 0.276 |
| WBC_m_ [/nl] | | 13.4 (10.6,16.3) | 13.1 (10.5,16) | 0.596 |
| Histology | |  |  |  |
| PDAC/CP | | 85 (54.1) | 83 (52.2) | 0.816 |
| other | | 72 (45.9) | 76 (47.8) |  |
| Type of resection | |  |  |  |
| Pancreatic head resection | | 138 (87.9) | 119 (74.8) | 0.005 |
| DPR | | 19 (12.1) | 40 (25.2) |  |
| PVR | | 12 (7.6) | 10 (6.3) | 0.801 |
| Pancreatic parenchyma | |  |  |  |
| soft | | 55 (35) | 52 (32.7) | 0.707 |
| hard | | 44 (28) | 41 (25.8) |  |
| unknown | | 58 (36.9) | 66 (41.5) |  |
| MPD | |  |  |  |
| ≤3mm | | 99 (63.1) | 94 (59.1) | 0.767 |
| >3mm | | 53 (33.8) | 59 (37.1) |  |
| unknown | | 5 (3.2) | 6 (3.8) |  |
| Bile duct stent | | 38 (24.2) | 27 (17) | 0.147 |
| Antibiotics preop | | 7 (4.5) | 2 (1.3) | 0.17 |
| Antibiotics 24h postop | | 76 (48.4) | 63 (39.6) | 0.144 |
| POPF B/C | | 61 (38.9) | 47 (29.6) | 0.105 |
| PPH B/C | | 36 (22.9) | 30 (18.9) | 0.453 |
| PPAP B/C | | 16 (10.2) | 12 (7.5) | 0.529 |
| Major complication rate CDC≥3b | | 54 (34.4) | 50 (31.4) | 0.661 |
| *Data are given as median (Q1, Q3) for continuous variables and as n (%) for categorical variables. Continuous variables were compared using the Kruskal-Wallis test and categorical variables using the Pearson χ² test.*  *Abbreviations: BMI – body mass index, DPR – distal pancreatectomy, CDC – Clavien-Dindo Classification, CP – chronic pancreatitis, CRP – C-reactive protein, h – hours, IL-6 – interleukin-6, __m_ – median, MPD – main pancreatic duct diameter, PDAC – pancreatic ductal adenocarcinoma, POPF – postoperative pancreatic fistula, PPAP – postpancreatectomy acute pancreatitis, PPH – postpancreatectomy hemorrhage, __pre_ – preoperative, PVR – portal vein resection, WBC – white blood cell count* | | | | |

**Table S3: Univariable analysis comparing median IL-6, Lipase and WBC count across categories of various clinical parameters**

| **Parameter** | | **IL-6 [pg/ml]** | | | **Lipase [U/l]** | | | **WBC [/nl]** | |
| --- | --- | --- | --- | --- | --- | --- | --- | --- | --- |
|  | Median (Q1, Q3) | | p | Median (Q1, Q3) | | p | Median (Q1, Q3) | | p |
| sex |  | |  |  | |  |  | |  |
| female | 202.2 (138.2, 366.4) | | 0.452 | 47.5 (9.5, 135.2) | | 0.077 | 13.8 (10.4, 16.7) | | 0.904 |
| male | 257 (131.4, 402.4) | |  | 74 (24.2, 145) | |  | 13.3 (10.7, 15.8) | |  |
| age |  | |  |  | |  |  | |  |
| ≤70 | 204.4 (125.5, 328) | | 0.003 | 67 (20, 146) | | 0.646 | 13.6 (10.6, 17.1) | | 0.639 |
| >70 | 311.5 (177.7, 547) | |  | 60 (15.6, 135) | |  | 13 (10.9, 15.7) | |  |
| BMI [kg/m²] |  | |  |  | |  |  | |  |
| ≤25 | 213 (137.8, 371.5) | | 0.804 | 54 (14, 111) | | 0.083 | 12.9 (10.6, 15.8) | | 0.181 |
| >25 | 247 (137, 385) | |  | 70.8 (25.4, 233) | |  | 13.8 (10.9, 18.2) | |  |
| histology |  | |  |  | |  |  | |  |
| PDAC/CP | 194.5 (119.5, 328) | | 0.004 | 25.5 (8.5, 68) | | <0.001 | 12.9 (10.6, 16.3) | | 0.691 |
| other | 268.9 (178.1, 483) | |  | 108.2 (64, 341.8) | |  | 13.7 (10.7, 16) | |  |
| type of resection |  | |  |  | |  |  | |  |
| Pancreatic head resection | 235 (156.1, 396.1) | | 0.004 | 64.5 (14, 185.8) | | 0.927 | 13.1 (10.5, 15.8) | | 0.014 |
| DPR | 125.5 (59.2, 240.6) | |  | 58 (43.5, 72.2) | |  | 16 (12.6, 19.4) | |  |
| splenectomy |  | |  |  | |  |  | |  |
| yes | 125.5 (54.2, 263.8) | | 0.011 | 56 (40, 70.5) | | 0.672 | 16.2 (12.3, 20.3) | | 0.014 |
| no | 232.9 (154.8, 391.4) | |  | 66.5 (14, 190) | |  | 13.2 (10.5, 15.7) | |  |
| PVR |  | |  |  | |  |  | |  |
| yes | 306.8 (174.9, 414) | | 0.411 | 38.2 (6.1, 87.5) | | 0.128 | 13 (8, 19.1) | | 0.625 |
| no | 227 (137.8, 370) | |  | 66 (17, 141) | |  | 13.3 (10.7, 16.2) | |  |
| MPD |  | |  |  | |  |  | |  |
| ≤3mm | 232.9 (149.2, 386.4) | | 0.766 | 74 (35, 238.5) | | <0.001 | 13.3 (10.7, 16.5) | | 0.608 |
| >3mm | 231.1 (119.5, 398.5) | |  | 20 (9, 78) | |  | 13.4 (10.3, 16.1) | |  |
| Bile duct stent |  | |  |  | |  |  | |  |
| yes | 301 (189.5, 513.7) | | 0.009 | 35.8 (10, 107.2) | | 0.070 | 12.4 (10.2, 14.8) | | 0.191 |
| no | 212 (127.4, 355.5) | |  | 69.5 (23.2, 145) | |  | 13.5 (10.8, 16.7) | |  |
| Antibiotics preop |  | |  |  | |  |  | |  |
| yes | 217.5 (198.2, 759.5) | | 0.529 | 54 (27.5, 91.5) | | 0.583 | 13.6 (11.5, 16.5) | | 0.604 |
| no | 229.9 (134.9, 380.6) | |  | 64.5 (16.2, 143.2) | |  | 13.3 (10.6, 16.2) | |  |
| Antibiotics 24h postop |  | |  |  | |  |  | |  |
| yes | 260.4 (159.8, 412.2) | | 0.161 | 53.5 (16, 130.1) | | 0.283 | 13.1 (10.6, 16.4) | | 0.861 |
| no | 212 (119.5, 358.4) | |  | 70 (17, 146) | |  | 13.4 (10.7, 16.1) | |  |
| POPF |  | |  |  | |  |  | |  |
| none/ Biochemical leak | 194.2 (117.5, 327.1) | | 0.001 | 34.2 (9, 89) | | <0.001 | 12.6 (9.9, 14.9) | | 0.002 |
| B/C | 309.1 (194.5, 460.5) | |  | 133 (58, 326) | |  | 13.9 (11.9, 19.2) | |  |
| PPH |  | |  |  | |  |  | |  |
| none/A | 226.2 (130, 366) | | 0.124 | 56.5 (14.5, 111) | | 0.009 | 13.1 (10.6, 16) | | 0.265 |
| B/C | 257 (175.7, 468.3) | |  | 117 (48, 459.1) | |  | 13.7 (11, 17.6) | |  |
| PPAP |  | |  |  | |  |  | |  |
| none/A | 226.2 (131, 366) | | 0.004 | n.a. | |  | 13.3 (10.6, 16.4) | | 0.593 |
| B/C | 372.5 (208.4, 931.6) | |  |  | |  | 13.8 (11.2, 16.2) | |  |
| complications |  | |  |  | |  |  | |  |
| none/ CDC<3b | 207 (128.2, 359.2) | | 0.022 | 59 (15.5, 111.2) | | 0.145 | 13.4 (10.8, 15.4) | | 0.975 |
| CDC≥3b | 279 (169.1, 529.2) | |  | 68.8 (21.4, 306.4) | |  | 13.1 (10.4, 17) | |  |
| Values are the per-patient median concentration within 48 hours after resection, given as median (Q1, Q3); subgroups were compared using the Kruskal-Wallis test. Serum lipase is not reported for PPAP B/C (n.a.), as PPAP was defined by postoperative lipase elevation.  Abbreviations: BMI – body mass index, DPR – distal pancreatectomy, CDC – Clavien-Dindo Classification, CP – chronic pancreatitis, IL-6 – interleukin-6, MPD – main pancreatic duct diameter, PDAC – pancreatic ductal adenocarcinoma, POPF – postoperative pancreatic fistula, PPAP – postpancreatectomy acute pancreatitis, PPH – postpancreatectomy hemorrhage, PVR – portal vein resection, WBC – white blood cell count | | | | | | | | | |

**Figure S1**: **Example of postoperative IL-6 kinetics in four representative patients.** Circles show measured values. Filled triangles show values reconstructed by linear interpolation within a patient's own measurement window; open triangles show values extrapolated beyond it using a linear mixed-effects model of log-transformed concentrations with a cubic time trend and patient-specific random intercept and slope. In leave-one-measurement-out validation, the median absolute percentage error was 24.3% within the measurement window and 45.6% and 33.0% before the first and after the last available measurement, respectively. The same approach was applied to serum lipase and white blood cell count.

**Table S4: Origin of the values at the analysed time points.** For each marker and time point, the table gives the number and proportion of the 157 patients whose value was measured, interpolated within the patient's own measurement window, or extrapolated before the first or after the last available measurement. At 42 hours, 78 % of IL-6 values would have had to be extrapolated beyond the last available measurement; this time point was therefore excluded from all further analyses.

| Marker | Time (h) | Measured n (%) | Interpolated n (%) | Extrapolated before first value, n (%) | Extrapolated after last value, n (%) |
| --- | --- | --- | --- | --- | --- |
| IL-6 | 6 | 0 (0) | 113 (72) | 43 (27) | 1 (1) |
|  | 12 | 2 (1) | 105 (67) | 30 (19) | 20 (13) |
|  | 18 | 0 (0) | 81 (52) | 24 (15) | 52 (33) |
|  | 24 | 1 (1) | 90 (57) | 14 (9) | 52 (33) |
|  | 30 | 0 (0) | 96 (61) | 7 (4) | 54 (34) |
|  | 36 | 1 (1) | 87 (55) | 1 (1) | 68 (43) |
|  | *42* | *0 (0)* | *35 (22)* | *0 (0)* | *122 (78)* |
| Lipase | 6 | 0 (0) | 132 (84) | 25 (16) | 0 (0) |
|  | 12 | 2 (1) | 133 (85) | 12 (8) | 10 (6) |
|  | 18 | 0 (0) | 129 (82) | 2 (1) | 26 (17) |
|  | 24 | 0 (0) | 128 (82) | 0 (0) | 29 (18) |
|  | 30 | 0 (0) | 127 (81) | 0 (0) | 30 (19) |
|  | 36 | 1 (1) | 96 (61) | 0 (0) | 60 (38) |
|  | *42* | *0 (0)* | *17 (11)* | *0 (0)* | *140 (89)* |
| WBC | 6 | 0 (0) | 139 (89) | 18 (11) | 0 (0) |
|  | 12 | 2 (1) | 139 (89) | 8 (5) | 8 (5) |
|  | 18 | 0 (0) | 135 (86) | 1 (1) | 21 (13) |
|  | 24 | 0 (0) | 135 (86) | 0 (0) | 22 (14) |
|  | 30 | 0 (0) | 134 (85) | 0 (0) | 23 (15) |
|  | 36 | 1 (1) | 122 (78) | 0 (0) | 34 (22) |
|  | *42* | *0 (0)* | *46 (29)* | *0 (0)* | *111 (71)* |
| Abbreviations: h – hours, IL-6 – interleukin-6, WBC – white blood cell count. | | | | | |

**Table S5: Accuracy of the reconstructed values.** Leave-one-measurement-out validation: each observed value was omitted in turn and reconstructed from the remaining data by linear interpolation within the patient's own measurement window and by the mixed-effects model beyond it. MdAPE, median absolute percentage error; bias, median ratio of predicted to observed value (1.00 = no systematic deviation).

| Marker | Position of the omitted value | n | MdAPE (%) | Bias |
| --- | --- | --- | --- | --- |
| IL-6 | Before first measurement | 101 | 45.6 | 0.99 |
|  | Within measurement window | 219 | 24.3 | 1.10 |
|  | After last measurement | 102 | 33.0 | 1.10 |
| Lipase | Before first measurement | 122 | 44.7 | 0.93 |
|  | Within measurement window | 203 | 29.1 | 1.02 |
|  | After last measurement | 123 | 29.0 | 1.07 |
| WBC | Before first measurement | 136 | 19.7 | 1.06 |
|  | Within measurement window | 311 | 10.6 | 1.00 |
|  | After last measurement | 136 | 10.6 | 0.98 |
| Abbreviations: IL-6 – interleukin-6, MdAPE – median absolute percentage error, WBC – white blood cell count. | | | | |

**Figure S2:** **Correlation of laboratory parameters within the first 48 hours after pancreatic resection (n = 157).** The heat map shows Spearman rank correlation coefficients; colour encodes direction and strength, and the coefficient is given in each cell. Numeric subscripts denote the postoperative time point in hours. Coefficients are descriptive and not adjusted for multiple comparisons. Minimum and maximum IL-6 values are shown for descriptive purposes only and were not used in any of the diagnostic or regression analyses.

Abbreviations: CRP – C-reactive protein, IL-6 – interleukin-6, max – maximum recorded value within the first 48 hours, min – minimum recorded value, pre – preoperative baseline, WBC – white blood cells count

**Figure S3:** **ROC curves and predictive performance of early postoperative biomarkers for POPF B/C.**

The panels illustrate the discriminative power of serial IL-6 [pg/ml], lipase[U/l], and WBC count [/nl] values within the first 48 hours post-resection. Numeric subscripts denote the specific postoperative time points in hours. The embedded table summarizes key diagnostic criteria (AUC, cut-off, sensitivity [%], specificity [%], PPV [%], and NPV [%]). Optimal cut-off was estimated with the “closest topleft” method. The reported cut-off is the median of these thresholds across 500 bootstrap samples, whereas sensitivity, specificity, PPV and NPV refer to the optimal threshold in the original data and are corrected for optimism arising from selecting the threshold in the same data.

| **Parameter** | **AUC (CI)** | **Cut-off** | **Sensitivity [%]** | **Specificity [%]** | **PPV [%]** | **NPV [%]** |
| --- | --- | --- | --- | --- | --- | --- |
| IL-6_6_ | 0.601 (0.512-0.691) | 348.5 pg/ml | 59 | 60 | 48 | 69 |
| IL-6_12_ | 0.617 (0.528-0.707) | 270.5 pg/ml | 55 | 65 | 50 | 69 |
| IL-6_18_ | 0.643 (0.553-0.732) | 260.0 pg/ml | 48 | 74 | 56 | 69 |
| IL-6_24_ | 0.661 (0.573-0.75) | 204.8 pg/ml | 52 | 66 | 50 | 68 |
| IL-6_30_ | 0.679 (0.593-0.766) | 190.2 pg/ml | 64 | 58 | 49 | 71 |
| IL-6_36_ | 0.715 (0.632-0.798) | 191.8 pg/ml | 70 | 59 | 52 | 76 |
| Lipase_6_ | 0.767 (0.691-0.843) | 82.7 U/l | 66 | 68 | 57 | 76 |
| Lipase_12_ | 0.777 (0.702-0.852) | 87.1 U/l | 68 | 72 | 61 | 78 |
| Lipase_18_ | 0.775 (0.701-0.85) | 71.8 U/l | 68 | 71 | 61 | 78 |
| Lipase_24_ | 0.755 (0.677-0.832) | 58.1 U/l | 66 | 69 | 58 | 76 |
| Lipase_30_ | 0.746 (0.668-0.824) | 47.7 U/l | 70 | 65 | 56 | 77 |
| Lipase_36_ | 0.757 (0.68-0.834) | 40.1 U/l | 81 | 58 | 55 | 83 |
| WBC_6_ | 0.644 (0.557-0.731) | 13.8 /nl | 60 | 53 | 45 | 68 |
| WBC_12_ | 0.612 (0.523-0.7) | 11.9 /nl | 71 | 45 | 45 | 71 |
| WBC_18_ | 0.624 (0.536-0.711) | 12.3 /nl | 65 | 46 | 43 | 68 |
| WBC_24_ | 0.628 (0.54-0.715) | 13.0 /nl | 71 | 40 | 43 | 70 |
| WBC_30_ | 0.626 (0.537-0.716) | 13.8 /nl | 55 | 52 | 42 | 65 |
| WBC_36_ | 0.623 (0.533-0.714) | 13.9 /nl | 59 | 52 | 44 | 67 |
| *Abbreviations: AUC – area under the curve, CI – confidence interval, IL-6 – interleukin-6, NPV – negative predictive value, POPF – postoperative pancreatic fistula, PPV – positive predictive value, ROC – Receiver Operating Characteristic Curve, WBC – white blood cell count* | | | | | |  |

**Figure S4:** **ROC curves and predictive performance of early postoperative biomarkers for PPAP B/C.** The panels illustrate the discriminative power of serial IL-6[pg/ml] and WBC[/nl] values within the first 48 hours post-resection. Numeric subscripts denote the specific postoperative time points in hours. The embedded table summarizes key diagnostic criteria (AUC, cut-off, sensitivity [%], specificity [%], PPV [%], and NPV [%]). Optimal cut-off was estimated with the “closest topleft” method. The reported cut-off is the median of these thresholds across 500 bootstrap samples, whereas sensitivity, specificity, PPV and NPV refer to the optimal threshold in the original data and are corrected for optimism arising from selecting the threshold in the same data.

| **Parameter** | **AUC (CI)** | **Cut-off** | **Sensitivity [%]** | **Specificity [%]** | **PPV [%]** | **NPV [%]** |
| --- | --- | --- | --- | --- | --- | --- |
| IL-6_6_ | 0.642 (0.505-0.78) | 347.8 pg/ml | 60 | 54 | 12 | 93 |
| IL-6_12_ | 0.710 (0.574-0.846) | 298.7 pg/ml | 63 | 67 | 18 | 94 |
| IL-6_18_ | 0.743 (0.607-0.878) | 301.2 pg/ml | 62 | 73 | 21 | 95 |
| IL-6_24_ | 0.782 (0.661-0.903) | 301.8 pg/ml | 61 | 77 | 23 | 95 |
| IL-6_30_ | 0.792 (0.667-0.916) | 326.0 pg/ml | 63 | 82 | 28 | 95 |
| IL-6_36_ | 0.800 (0.685-0.916) | 296.1 pg/ml | 62 | 80 | 26 | 95 |
| WBC_6_ | 0.571 (0.415-0.727) | 13.9 /nl | 56 | 55 | 12 | 92 |
| WBC_12_ | 0.522 (0.376-0.668) | 12.5 /nl | 68 | 40 | 11 | 92 |
| WBC_18_ | 0.540 (0.396-0.683) | 13.2 /nl | 48 | 51 | 10 | 90 |
| WBC_24_ | 0.569 (0.431-0.707) | 13.8 /nl | 55 | 52 | 11 | 91 |
| WBC_30_ | 0.538 (0.386-0.689) | 15.3 /nl | 44 | 66 | 13 | 91 |
| WBC_36_ | 0.494 (0.333-0.655) | 15.2 /nl | 45 | 63 | 12 | 91 |
| *Abbreviations: AUC – area under the curve, CI – confidence interval, IL-6 – interleukin-6, NPV – negative predictive value, PPAP – postpancreatectomy acute pancreatitis, PPV – positive predictive value, ROC – Receiver Operating Characteristic Curve, WBC – white blood cell count* | | | | | | |

**Figure S5:** **ROC curves and predictive performance of early postoperative biomarkers for major complications Clavien-Dindo grade ≥ 3b.** The panels illustrate the discriminative power of serial IL-6 [pg/ml], lipase[U/l], and WBC [/nl] values within the first 48 hours post-resection. Numeric subscripts denote the specific postoperative time points in hours. The embedded table summarizes key diagnostic criteria (AUC, cut-off, sensitivity [%], specificity [%], PPV [%], and NPV [%]). Optimal cut-off was estimated with the “closest topleft” method. The reported cut-off is the median of these thresholds across 500 bootstrap samples, whereas sensitivity, specificity, PPV and NPV refer to the optimal threshold in the original data and are corrected for optimism arising from selecting the threshold in the same data.

| **Parameter** | **AUC (CI)** | **Cut-off** | **Sensitivity [%]** | **Specificity [%]** | **PPV [%]** | **NPV [%]** |
| --- | --- | --- | --- | --- | --- | --- |
| IL-6_6_ | 0.602 (0.508-0.696) | 352.7 pg/ml | 53 | 58 | 40 | 70 |
| IL-6_12_ | 0.603 (0.507-0.698) | 258.9 pg/ml | 58 | 59 | 43 | 73 |
| IL-6_18_ | 0.607 (0.51-0.705) | 221.5 pg/ml | 61 | 52 | 40 | 72 |
| IL-6_24_ | 0.61 (0.511-0.709) | 208.1 pg/ml | 56 | 59 | 42 | 72 |
| IL-6_30_ | 0.616 (0.518-0.714) | 191.9 pg/ml | 64 | 57 | 44 | 75 |
| IL-6_36_ | 0.636 (0.539-0.733) | 208.3 pg/ml | 55 | 62 | 44 | 73 |
| Lipase_6_ | 0.564 (0.467-0.66) | 85.2 U/l | 46 | 57 | 36 | 67 |
| Lipase_12_ | 0.554 (0.458-0.651) | 118.8 U/l | 39 | 69 | 41 | 68 |
| Lipase_18_ | 0.578 (0.481-0.674) | 88.4 U/l | 47 | 62 | 40 | 69 |
| Lipase_24_ | 0.591 (0.495-0.688) | 85.2 U/l | 47 | 67 | 43 | 70 |
| Lipase_30_ | 0.607 (0.511-0.702) | 55.5 U/l | 50 | 62 | 41 | 70 |
| Lipase_36_ | 0.589 (0.495-0.684) | 45.6 U/l | 57 | 62 | 44 | 73 |
| WBC_6_ | 0.525 (0.423-0.627) | 14.3 /nl | 45 | 57 | 35 | 66 |
| WBC_12_ | 0.482 (0.38-0.585) | 14.3 /nl | 42 | 58 | 34 | 65 |
| WBC_18_ | 0.498 (0.397-0.599) | 14.2 /nl | 47 | 50 | 33 | 64 |
| WBC_24_ | 0.534 (0.434-0.634) | 14.6 /nl | 42 | 66 | 40 | 68 |
| WBC_30_ | 0.539 (0.438-0.639) | 14.7 /nl | 46 | 66 | 41 | 70 |
| WBC_36_ | 0.537 (0.436-0.637) | 15.7 /nl | 46 | 71 | 46 | 71 |
| *Abbreviations: AUC – area under the curve, CI – confidence interval, IL-6 – interleukin-6, NPV – negative predictive value PPV – positive predictive value, ROC – Receiver Operating Characteristic Curve, WBC – white blood cell count* | | | | | | |

**Table S6:** **Comparison of apparent and bootstrap optimism-corrected predictive metrics for POPF B/C, PPAP B/C and major postoperative complications Clavien-Dindo grade ≥ 3b.** The table displays the apparent and optimism-corrected AUC, sensitivity [%], specificity [%], PPV [%], and NPV [%] for IL-6 [pg/ml], lipase [U/l] and WBC count [/nl] within the first 48 hours post-resection. Numeric subscripts denote the specific postoperative time points (in hours). The table shows also the optimal cut-off for the original data and the median cut-off across all bootstrap sets.

| Parameter | AUC apparent | AUC corrected | Cut-off | Cut-off median | Sens a, % | Sens c, % | Spec a, % | Spec c, % | PPV a, % | PPV c, % | NPV a, % | NPV c, % |
| --- | --- | --- | --- | --- | --- | --- | --- | --- | --- | --- | --- | --- |
| **Outcome: POPF B/C** | | | | | | | | | | | | |
| IL-6₆ [pg/ml] | 0.599 | 0.601 | 348.5 | 348.5 | 61 | 59 | 61 | 60 | 50 | 48 | 71 | 69 |
| IL-6₁₂ [pg/ml] | 0.614 | 0.617 | 272.0 | 270.5 | 57 | 55 | 67 | 65 | 52 | 50 | 71 | 69 |
| IL-6₁₈ [pg/ml] | 0.640 | 0.643 | 272.4 | 260.0 | 51 | 48 | 76 | 74 | 57 | 56 | 71 | 69 |
| IL-6₂₄ [pg/ml] | 0.659 | 0.661 | 224.4 | 204.8 | 56 | 52 | 68 | 66 | 52 | 50 | 71 | 68 |
| IL-6₃₀ [pg/ml] | 0.676 | 0.679 | 190.0 | 190.2 | 67 | 64 | 59 | 58 | 51 | 49 | 74 | 71 |
| IL-6₃₆ [pg/ml] | 0.713 | 0.715 | 175.9 | 191.8 | 74 | 70 | 60 | 59 | 54 | 52 | 78 | 76 |
| Lipase₆ [U/l] | 0.765 | 0.767 | 82.7 | 82.7 | 69 | 66 | 70 | 68 | 59 | 57 | 78 | 76 |
| Lipase₁₂ [U/l] | 0.776 | 0.777 | 82.9 | 87.1 | 70 | 68 | 73 | 72 | 62 | 61 | 80 | 78 |
| Lipase₁₈ [U/l] | 0.774 | 0.775 | 74.0 | 71.8 | 70 | 68 | 73 | 71 | 62 | 61 | 80 | 78 |
| Lipase₂₄ [U/l] | 0.752 | 0.755 | 63.8 | 58.1 | 69 | 66 | 71 | 69 | 60 | 58 | 78 | 76 |
| Lipase₃₀ [U/l] | 0.744 | 0.746 | 48.4 | 47.7 | 72 | 70 | 67 | 65 | 58 | 56 | 79 | 77 |
| Lipase₃₆ [U/l] | 0.755 | 0.757 | 28.2 | 40.1 | 84 | 81 | 59 | 58 | 57 | 55 | 85 | 83 |
| WBC₆ [/nl] | 0.648 | 0.644 | 13.3 | 13.8 | 64 | 60 | 55 | 53 | 48 | 45 | 71 | 68 |
| WBC₁₂ [/nl] | 0.613 | 0.612 | 11.9 | 11.9 | 74 | 71 | 47 | 45 | 47 | 45 | 74 | 71 |
| WBC₁₈ [/nl] | 0.625 | 0.624 | 12.3 | 12.3 | 69 | 65 | 48 | 46 | 46 | 43 | 71 | 68 |
| WBC₂₄ [/nl] | 0.629 | 0.628 | 11.8 | 13.0 | 75 | 71 | 43 | 40 | 46 | 43 | 73 | 70 |
| WBC₃₀ [/nl] | 0.628 | 0.626 | 13.3 | 13.8 | 59 | 55 | 54 | 52 | 45 | 42 | 68 | 65 |
| WBC₃₆ [/nl] | 0.625 | 0.623 | 13.2 | 13.9 | 62 | 59 | 54 | 52 | 46 | 44 | 69 | 67 |
| **Outcome: PPAP B/C** | | | | | | | | | | | | |
| IL-6₆ [pg/ml] | 0.641 | 0.642 | 345.9 | 347.8 | 69 | 60 | 55 | 54 | 15 | 12 | 94 | 93 |
| IL-6₁₂ [pg/ml] | 0.708 | 0.710 | 301.5 | 298.7 | 69 | 63 | 68 | 67 | 20 | 18 | 95 | 94 |
| IL-6₁₈ [pg/ml] | 0.742 | 0.743 | 285.6 | 301.2 | 69 | 62 | 74 | 73 | 23 | 21 | 95 | 95 |
| IL-6₂₄ [pg/ml] | 0.781 | 0.782 | 293.1 | 301.8 | 69 | 61 | 78 | 77 | 26 | 23 | 96 | 95 |
| IL-6₃₀ [pg/ml] | 0.790 | 0.792 | 329.8 | 326.0 | 69 | 63 | 82 | 82 | 31 | 28 | 96 | 95 |
| IL-6₃₆ [pg/ml] | 0.798 | 0.800 | 296.2 | 296.1 | 69 | 62 | 80 | 80 | 28 | 26 | 96 | 95 |
| WBC₆ [/nl] | 0.572 | 0.571 | 13.9 | 13.9 | 62 | 56 | 56 | 55 | 14 | 12 | 93 | 92 |
| WBC₁₂ [/nl] | 0.522 | 0.522 | 11.9 | 12.5 | 75 | 68 | 40 | 40 | 12 | 11 | 93 | 92 |
| WBC₁₈ [/nl] | 0.540 | 0.540 | 13.2 | 13.2 | 56 | 48 | 52 | 51 | 12 | 10 | 91 | 90 |
| WBC₂₄ [/nl] | 0.570 | 0.569 | 13.4 | 13.8 | 62 | 55 | 52 | 52 | 13 | 11 | 92 | 91 |
| WBC₃₀ [/nl] | 0.541 | 0.538 | 15.3 | 15.3 | 50 | 44 | 67 | 66 | 15 | 13 | 92 | 91 |
| WBC₃₆ [/nl] | 0.497 | 0.494 | 15.2 | 15.2 | 50 | 45 | 63 | 63 | 13 | 12 | 92 | 91 |
| **Outcome: Major complications, Clavien-Dindo grade ≥ 3b** | | | | | | | | | | | | |
| IL-6₆ [pg/ml] | 0.601 | 0.602 | 350.4 | 352.7 | 57 | 53 | 59 | 58 | 42 | 40 | 73 | 70 |
| IL-6₁₂ [pg/ml] | 0.605 | 0.603 | 256.6 | 258.9 | 61 | 58 | 61 | 59 | 45 | 43 | 75 | 73 |
| IL-6₁₈ [pg/ml] | 0.611 | 0.607 | 210.6 | 221.5 | 65 | 61 | 54 | 52 | 43 | 40 | 75 | 72 |
| IL-6₂₄ [pg/ml] | 0.611 | 0.610 | 211.4 | 208.1 | 59 | 56 | 61 | 59 | 44 | 42 | 74 | 72 |
| IL-6₃₀ [pg/ml] | 0.617 | 0.616 | 192.4 | 191.9 | 67 | 64 | 58 | 57 | 46 | 44 | 77 | 75 |
| IL-6₃₆ [pg/ml] | 0.636 | 0.636 | 203.2 | 208.3 | 59 | 55 | 64 | 62 | 46 | 44 | 75 | 73 |
| Lipase₆ [U/l] | 0.566 | 0.564 | 86.4 | 85.2 | 50 | 46 | 59 | 57 | 39 | 36 | 69 | 67 |
| Lipase₁₂ [U/l] | 0.557 | 0.554 | 144.1 | 118.8 | 43 | 39 | 71 | 69 | 43 | 41 | 70 | 68 |
| Lipase₁₈ [U/l] | 0.580 | 0.578 | 89.0 | 88.4 | 50 | 47 | 64 | 62 | 42 | 40 | 71 | 69 |
| Lipase₂₄ [U/l] | 0.595 | 0.591 | 85.2 | 85.2 | 50 | 47 | 69 | 67 | 46 | 43 | 72 | 70 |
| Lipase₃₀ [U/l] | 0.609 | 0.607 | 56.1 | 55.5 | 54 | 50 | 64 | 62 | 44 | 41 | 73 | 70 |
| Lipase₃₆ [U/l] | 0.593 | 0.589 | 45.6 | 45.6 | 59 | 57 | 63 | 62 | 46 | 44 | 75 | 73 |
| WBC₆ [/nl] | 0.528 | 0.525 | 14.2 | 14.3 | 48 | 45 | 59 | 57 | 38 | 35 | 69 | 66 |
| WBC₁₂ [/nl] | 0.484 | 0.482 | 14.1 | 14.3 | 44 | 42 | 59 | 58 | 36 | 34 | 67 | 65 |
| WBC₁₈ [/nl] | 0.499 | 0.498 | 13.2 | 14.2 | 50 | 47 | 51 | 50 | 35 | 33 | 66 | 64 |
| WBC₂₄ [/nl] | 0.535 | 0.534 | 14.8 | 14.6 | 44 | 42 | 68 | 66 | 42 | 40 | 70 | 68 |
| WBC₃₀ [/nl] | 0.541 | 0.539 | 14.7 | 14.7 | 48 | 46 | 67 | 66 | 43 | 41 | 71 | 70 |
| WBC₃₆ [/nl] | 0.539 | 0.537 | 15.7 | 15.7 | 48 | 46 | 72 | 71 | 47 | 46 | 73 | 71 |
| a, apparent value in the original data; c, corrected for optimism by bootstrapping. Cut-off is the optimal threshold in the original data (closest-topleft criterion), cut-off median the median of these thresholds across the bootstrap samples. | | | | | | | | | | | | |
| Abbreviations: AUC – area under the curve, IL-6 – interleukin-6, NPV – negative predictive value, POPF – postoperative pancreatic fistula, PPAP – postpancreatectomy acute pancreatitis, PPV – positive predictive value, WBC – white blood cell count. | | | | | | | | | | | | |
